# Mortality was predicted by depression and functional dependence in a cohort of elderly adults of Italian descent from southern Brazil

**DOI:** 10.1038/s41598-023-32617-1

**Published:** 2023-04-03

**Authors:** Emeline Pessin, Sandra C. Fuchs, Neide M. Bruscato, Felipe C. Fuchs, Emilio H. Moriguchi

**Affiliations:** 1 Postgraduate Program in Cardiology, School of Medicine, Universidade Federal do Rio Grande do Sul, Porto Alegre, RS Brazil; 2 Clinical Research Center, INCT PREVER, Hospital de Clínicas de Porto Alegre, Ramiro Barcelos, 2350, Porto Alegre, RS 90035-903 Brazil; 3 Division of Cardiology, Hospital de Clínicas de Porto Alegre, Av. Protásio Alves, no. 211, Santa Cecilia, Porto Alegre, RS 90035-903 Brazil; 4 Clinical Research Center, INCT PREVER, CPC, 5º and Hospital de Clínicas de Porto Alegre, Ramiro Barcelos, 2350, Porto Alegre, RS 90035-903 Brazil

**Keywords:** Cardiovascular biology, Risk factors, Depression

## Abstract

The older population has an increasing burden of non-communicable disease, which can potentially be associated with physical and mental disabilities and shorten life spam. To investigate whether depression, loss of functionality for activities of daily living, and lower social support are associated with all-cause mortality in the older population of Italian descent. This population-based cohort study was conducted in Veranópolis, a country city from southern Brazil, among individuals aged 60 years or older. Interviews were performed in a systematic random sampling regarding demographic, socioeconomic, and psychosocial variables, in addition to depression (Geriatric Depression Scale), activities of daily living (Barthel Index), and social support (Medical Outcomes Study scale). In the follow-up, participants were reinterviewed or, in case of death, the next of kin, and hospital records were revised. Hierarchical analysis was used to determine characteristics independently associated with all-cause mortality, using Poisson regression with robust variance, expressed as relative risk with 95% confidence intervals (RR; 95%CI). A total of 997 participants were enrolled and 882 participants completed the study, after 7.24 ± 2.41 years; with 581 remaining alive. The mean age was 73.12 ± 8.03 years, 4% were nonagenarians or centennials, and 62% were women. Symptoms of depression (RR: 1.04; 1.01–1.06) and functional dependence for ADL (RR: 1.00; 0.99–1.00) were associated with all-cause mortality, even after controlling for confounding factors. Lower social support was not associated with mortality (RR: 1.00; 0.99–1.01). Depression and functional dependence are independent predictors of all-cause mortality in the older population from Italian descent.

## Introduction

Cardiovascular disease is the main cause of disability and mortality worldwide^1^, leading to early loss of years of productive life^2^. Persistent exposures to elevated blood pressure^3^, hyperglycemia^4^, and smoking^5^ increase the risk of death in older adults, while aerobic activity, strength training, or combined exercise seem to reduce this risk^6^. Whether alcoholic beverage intake is a risk factor^7^ or affords cardiovascular protection^8,9^ remains controversial.

Veranópolis, a town located in the highlands of southern Brazil, has become known for the longevity of its population, which has the highest life expectancy in the country. Cohort studies of very old^10^ and older^11^ individuals have confirmed associations of cardiovascular risk factors (such as high blood pressure, extremes of sleep duration, and dyslipidemia) with mortality. Studies have also shown that reduced feelings of well-being, lack of purpose in life, hopelessness, and loneliness also increased the risk of mortality^12,13^. The older population, mostly of Italian descent, seems to share a social cohesion, enhancing the feeling of belonging to the community.

Multimorbidity is highly prevalent in older adults^14^, and those with effective social interactions seem to be more likely to survive than those with absent or disrupted relationships. The lack of social connections and isolation seem to have an effect comparable to that of traditional risk factors^15^, which may be even more relevant in older individuals due to the concomitant occurrence of frailty, functional dependence, and depression^16,17^. Although the mechanisms are not fully established, the effect of these risk factors when unmitigated by full social connections and socioeconomic status compatible with one’s needs should be investigated.

This city was founded by Italian immigrants who came to Brazil during the immigration period. Part of the population are bilingual, speaks Portuguese and an Italian dialect, and maintains traditions of closeness and friendly relationships. Given the dearth of studies on this topic conducted in the older population from middle-income countries, this cohort study was designed to investigate independent associations of depression, functionality for activities of daily living, and lower social support with all-cause mortality in the population into their sixties and beyond.

## Methods

### Study design

This population-based cohort study included individuals aged 60 years or older living in the town of Veranópolis, southern Brazil, certified as one of the Age-friendly cities and communities by the World Health Organization. Participants were selected through a systematic random sampling of all individuals aged 60 years or older residing in the municipality. The only exclusion criterion would be not residing in the municipality.

During 2009, enrolled participants were interviewed regarding characteristics potentially associated with the decision to have the influenza vaccine, as well as past medical history and demographic, socioeconomic, and psychosocial variables. Participants were selected by systematic random sampling from a record of all individuals aged 60 years or older living in the municipality. During the follow-up visit, participants were assessed between June 2017 and March 2018. Throughout this period, the investigators sought to locate all participants interviewed at baseline through telephone calls, home visits, contacting neighbors or the next of kin informed in the application, and making on-site visits to any secondary or additional addresses which participants might have provided in the hospital record.

Participants were scheduled to undergo an interview, as well as measurements and specimen collection for laboratory tests, at the study headquarters or at home. The next of kin of participants who had died between baseline and follow-up were contacted to obtain information through a verbal autopsy. All experiments were performed in accordance with relevant guidelines and regulations, and that the STROBE (Strengthening the Reporting of Observational Studies in Epidemiology) guidelines were followed. This study was approved by the Ethics Committee of Hospital de Clinicas de Porto Alegre (GPPG number: 170241), which is accredited by the Office of Human Research Protections as an Institutional Review Board, and registered at Plataforma Brasil. Written informed consent was provided from all participants or their next of kin.

### Studied variables

Vital status (dead or alive), determined through death certificates, was the primary clinical outcome. A verbal autopsy with the next of kin, review of hospital records, and interviews with attending physicians were used to outline the events surrounding death. All deaths that occurred between January 1, 2009 and March 31, 2018 were identified.

The exposures of interest included:

- Depression, investigated through the Geriatric Depression Scale (GDS), which consists of 15 items to measure how the respondent has been feeling^18^. This quantitative scale, used for screening, has been validated in Brazil; scores ≥ 5 are indicative of moderate depression, 1–4 of mild depression, and < 5 of no depression^19^.
- Functionality for basic activities of daily living, determined by the 10 items of the Barthel Index, assessed self-care (feeding, transferring from chair to bed, grooming, toilet use, bathing, dressing, and sphincter control) and mobility (climbing/descending stairs and walking)^20^. This quantitative scale, ranging from zero to 100, was administered and categorized to identify individuals as totally independent (100 points) or dependent (< 100) for least one ADL.
- Social support, investigated using the Medical Outcomes Study (MOS) scale^21^, conceived to measure material social support, involving the provision of practical resources and material help; affective support, relating to demonstrations of love and affection; positive social interactions, i.e., having people to relax with or have fun with; emotional support, encompassing the social network’s ability to satisfy individual needs in relation to emotional problems; and, finally, an information dimension, which measures access to people on whom one can rely for advice, information, or guidance^22^. Answers were categorized as: never, rarely, sometimes, nearly always, and always, and added together to score from 19 to 95. The 80th percentile (scores > 88) was also used to identify lower social support.

Variables considered potential risk or protection factors were: sex, age (categorized as 60–69, 70–79, 80–102 years), education (years at school; categorized as 0–4, 5–8, and ≥ 9), having a partner (or spouse), retirement, self-reported regular physical activity, smoking status (categorized as never smoker, former smoker, or current smoker), alcohol intake (categorized as abusive consumption if daily intake ≥ 2 units for men or ≥ 1 unit for women, of beer (can), wine (glass), or spirits (dose); social consumption for a daily intake of < 2 or < 1 unit, respectively; abstemious otherwise), participation in group activities (religious group, community association, cultural group, political party, regular meeting with friends, sports team; categorized as present for those who were involved in at least one group), and previous diagnosis of heart disease or cancer.

The research team was composed of health professionals who were trained and supervised during the performance of standardized interviews, using a previously tested questionnaire^10^, and when administering the study scales.

### Sample size calculation and statistical analysis

Sample size calculation was based on the association between Barthel Index and mortality, assuming that functionally dependent older individuals would have a 50% risk of dying during the follow-up period versus 30% in those who were independent, considering an exposed–to-unexposed ratio of 12:9 and a 95% confidence level (95%CI). A sample of 800 participants would be needed to ensure statistical power > 95% (EPIDAT; PAHO, version 3.1).

**Figure 1:**
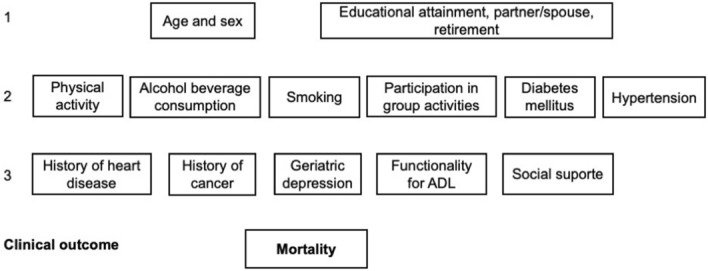
Hierarchical model of factors associated with mortality in older adults.

Analysis of associations, with calculation of relative risk (RR) and respective 95% confidence intervals, was performed through Poisson regression with robust variance. A hierarchical analysis^23^ was used to plan and conduct multivariate analysis, including the variables of interest (depression, functionality for ADL, and social support) and potential confounding factors (age, sex, education, marital status, retirement, regular physical activity, smoking, harmful alcohol use, participation in group activities, physician diagnosis of hypertension, diabetes mellitus, heart disease, and history of cancer). Figure 1 illustrates these sets of variables, starting from those temporally most distal to mortality—demographic (sex and age) and socioeconomic (education, marital status, and retirement)^23^. In an attempt to obtain a parsimonious model, a univariate analysis of risk factors potentially associated with mortality was carried out in each set, and the most strongly associated variables (P < 0.20) selected. Chi-square test of independence was used to determine if there was a statistically significant association between nominal variables and mortality. Risk factors that remained associated were carried forward into the multivariate analysis as confounding factors for the set itself and of subsequent sets. Depression, functional independence for ADL, and social support were analyzed adjusting for confounding factors within the set at the same level and at hierarchically superior ones^23^. The selected variables were categorized (to greater applicability of the results) and to allow better model fit on multivariate analysis. All statistical analyses were carried out in IBM SPSS Statistics for Windows, Version 21.0 (IBM Corp., Armonk, NY). Spearman’s correlation was used to test for correlation between the variables of interest on their original scales.

## Results

**Figure 2:**
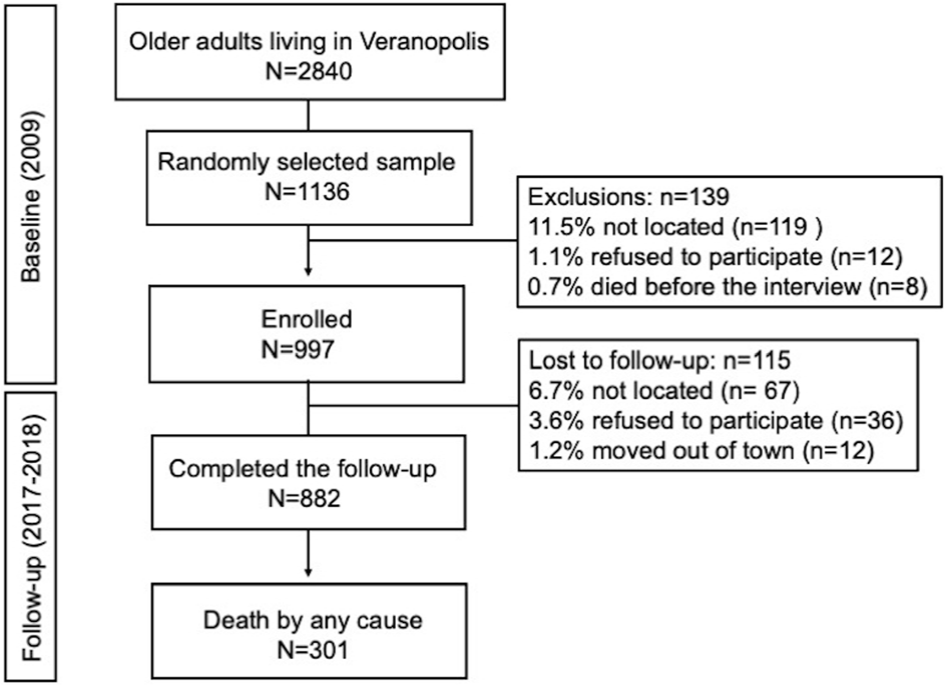
Flow diagram of study population.

Among participants potentially eligible at baseline, 10.48% were not found, 1.06% refused to participate, and 0.70% died before they could be interviewed, resulting in 997 participants enrolled (Fig. 2). After 7.24 ± 2.41 years of follow-up, 882 (88.46%) were interviewed and 115 (11.53%) had been lost: 67 were not found, 36 refused to participate, and 12 moved out of town.

Table 1 shows that participants were aged 73.1 ± 8.0 years on average. The majority were women, with low educational attainment, retired, and living with a partner. Most participants were non-smokers; more than half consumed alcoholic beverages and engaged in physical activity on a daily basis. Regarding previous diagnoses, 56.46% reported hypertension, 14.29% diabetes mellitus, 32.43% heart disease, and 5.82% cancer. Approximately one-third had depression, 83.90% were independent for ADLs and most had some type of social support.

**Table 1:** Characteristics associated with mortality in older adults (n = 882).

	Total N (%)	All-cause mortality	
		N (%)	*P*-value
Sex			0.015
Female	544 (61.67)	169 (31.06)	
Male	338 (38.32)	132 (39.05)	
Age (years)			< 0.001
60–69	335 (37.98)	39 (11.64)	
70–79	357 (40.48)	126 (35.29)	
80–89	155 (17.57)	136 (71.6)	
90–102	35 (4.00)	35 (100.00)	
Educational attainment (years)			0.002
≥ 9	127 (14.39)	34 (26.77)	
5–8	311 (35.26)	91 (29.26)	
0–4	444 (50.34)	176 (39.64)	
Partner			< 0.001
Yes	566 (64.17)	148 (26.14)	
No	316 (35.83)	153 (48.42)	
Retirement			< 0.001
Yes	802 (90.93)	289 (36.03)	
No	80 (9.07)	12 (15.00)	
Regular physical activity			< 0.001
No	384 (43.54)	162 (42.19)	
Yes	497 (56.34)	138 (27.76)	
Smoking			0.003
Never smoked	656 (74.38)	205 (31.25)	
Former smoker	190 (21.54)	77 (40.53)	
Current smoker	36 (4.08)	19 (52.78)	
Alcohol use			0.001
Abstemious	426 (48.30)	171 (40.14)	
Social	385 (43.65)	109 (28.31)	
Harmful	71 (8.04)	21 (29.58)	
Participation in group activities			< 0.001
No	368 (41.72)	173 (47.01)	
Yes	513 (58.16)	127 (24.76)	
Hypertension			0.02
No	384 (43.54)	115 (29.94)	
Yes	498 (56.46)	186 (37.34)	
Diabetes mellitus			0.803
No	756 (85.71)	257 (33.99)	
Yes	126 (14.29)	44 (34.92)	
Depression			0.004
No	631 (71.54)	196 (31.06)	
Mild	225 (25.51)	91 (40.44)	
Moderate/severe	26 (2.95)	14 (53.84)	
Functionality for ADL^b^			< 0.001
Independent	740 (83.90)	228 (30.81)	
Dependent	142 (16.09)	73 (51.41)	
Low social support^a^			0.204
No	172 (19.50)	66 (38.37)	
Yes	709 (80.39)	234 (33.00)	
Heart disease			
No	596 (67.57)	170 (28.52)	< 0.001
Yes	286 (32.43)	131 (45.80)	
Cancer			0.001
No	831 (94.22)	273 (32.85)	
Yes	51 (5.82)	28 (54.90)	

^a^Low social support: < 80th percentile on MOS score.

^b^Functional independence: Barthel Index = 100.

During the follow-up period, 301 (34.13%) participants died, mostly women, with low education level, at a mean age of 78.72 ± 8.14 years. There was an inverse association between educational attainment and mortality, as well as lower risk among individuals who were not retired and lived with a partner (Table 1). Considering lifestyle characteristics, individuals who participated in one or more group activities had lower risk of dying, but reduced social support was not associated with mortality. An increased risk of death was observed among smokers, those who self-reported as abstainers, and those with hypertension, heart disease, or a history of cancer. Depression, present in about one-third of the sample, was directly related to the risk of death; increasing depression severity was associated with higher mortality. A supplementary Table presents the studied sample and participants loose to follow-up.

As shown in Table 2, sex and age were strongly associated with mortality, even after controlling for biological confounding factors. Among socioeconomic characteristics, not having a partner remained as an independent risk factor, but educational attainment and retirement lost statistical significance after controlling for biological and socioeconomic variables. Among lifestyle characteristics, smoking remained associated with mortality even after taking into account traditional risk factors, while consumption of alcoholic beverages and participation in group activities were protective factors. Previous diagnosis of heart disease or cancer was an independent risk factor for mortality, even after controlling for multiple traditional risk factors. On univariate analysis, the risk of mortality increased 8% per additional point on the GDS score, decreasing to 4% in the model adjusted for biological, socioeconomic, lifestyle factors, comorbidity, and loss of functionality. Lower scores on the Barthel Index suggested greater risk of dying, and controlling for other risk factors did not substantially reduce the risk. Lower social support was not associated with mortality.

**Table 2:** Relative risk (95%CI) for mortality in the presence of traditional and neglected risk factors at baseline (n = 882).

	RR (95%CI)^a^	*P*-value	RR (95%CI)^b^	*P*-value
Sex		0.01		0.004*
Female	1.00		1.00	
Male	1.08 (1.05–1.51)		1.27 (1.08–1.50)	
Age (years)		< 0.001		< 0.001*
60–69	1.00		1.00	
70–79	3.03 (2.19–4.20)		3.01 (2.17–4.17)	
80–90	5.60 (4.08–7.68)		5.54 (4.05–7.60)	
90–102	8.59 (6.40–11.54)		8.84 (6.60–11.84)	
Partner		< 0.001		< 0.001**
Yes	1.00		1.00	
No	1.85 (1.55–2.22)		1.48 (1.22–1.78)	
Educational attainment (years)	0.95 (0.92–0.98)	0.002	0.98 (0.95–1.00)	0.11**
Regular physical activity		< 0.001		0.09***
Yes	1.00		1.00	
No	1.52 (1.26–1.83)		1.16 (0.98–1.38)	
Smoking		0.001		0.001***
Never smoked	1.00		1.00	
Former smoker	1.30 (1.06–1.59)		1.29 (1.02–1.63)	
Current smoker	1.69 (1.22–2.35)		1.82 (1.31–2.52)	
Alcohol use		0.001		0.001***
Abstemious	1.00		1.00	
Social	0.71 (0.58–1.07)		0.76 (0.64–0.90)	
Harmful	0.74 (0.51–0.92)		0.64 (0.45–0.92)	
Participation in group activities				
No	1.00	< 0.001	1.00	< 0.001
Yes	0.53 (0.44–0.63)		0.73 (0.61–0.86)	
Hypertension		0.02		0.143****
No	1.00		1.00	
Yes	1.25 (1.03–1.51)		1.14 (0.96–1.35)	
Diabetes mellitus		0.8		0.172****
No	1.00		1.00	
Yes	1.03 (0.79–1.33)		1.18 (0.93–1.50)	
Heart disease		< 0.001		0.007*****
No	1.00		1.00	
Yes	1.55 (1.30–1.86)		1.26 (1.07–1.49)	
Cancer		< 0.001		< 0.001*****
No	1.00		1.00	
Yes	1.67 (1.28–2.18)		1.86 (1.43–2.42)	
Depression scale	1.08 (1.05–1.11)	< 0.001	1.04 (1.01–1.06)	0.005*****
Barthel Index	0.98 (0.98–0.99)	< 0.001	0.996 (0.99–0.99)	0.032*****
Social support scale	1.00 (0.99–1.01)	0.7	1.00 (0.99–1.01)	0.801*****

^a^Relative risk and 95%CI without controlling for confounding factors.

^b^Relative risk and 95%CI after controlling for the following confounding factors:

*Model 1: age and sex.

**Model 2: model 1 + educational attainment and partner.

***Model 3: model 2 + physical activity, smoking, alcohol abuse, participation in group activities, and hypertension.

****Model 4: model 3 + history of heart disease, history of cancer, depression scale.

*****Model 5: model 4 + Barthel Index.

Table 3 shows correlations between the GDS, the Barthel Index, and the social support scale. All dimensions of social support were found to be negatively correlated with the depression scale. This suggests that the more material, affective, emotional, informational, and social interaction support that older people receive, the lower their score on the GDS. However, there was no statistically significant correlation between the Barthel Index and social support.

**Table 3:** Correlation between the depression scale, Barthel Index, and dimensions of social support (*ρ* and P-value).

	Depression scale	Barthel Index
Depression scale	–	− 0.19
		P < 0.001
Social support scale	− 0.23	0.02
	P < 0.001	P = 0.60
Material support dimension	− 0.14	0.06
	P < 0.001	P = 0.08
Affective support dimension	− 0.18	0.06
	P < 0.001	P = 0.07
Emotional support dimension	− 0.14	0.02
	P < 0.001	P = 0.60
Information support dimension	− 0.16	0.04
	P < 0.001	P = 0.20
Social interaction support dimension	− 0.25	0.008
	P < 0.001	P = 0.80

## Discussion

In this cohort study representative of a population aged 60–102 years, mostly Italian descent, symptoms of depression and loss of functionality for ADL were associated with higher mortality, even after controlling for the effect of traditional risk and protective factors. Older adults with symptoms of depression are subject to decreased implementation of self-care, adherence to medications, systematic medical monitoring, and healthy lifestyle habits, as well as less likely to adopt protective behaviors (such as participation in group activities, moderate intake of alcoholic beverages, and regular physical activity), but to increased time spent sitting or lying down. All-cause mortality can be explained by a set of risk exposures^8^, insufficiently mitigated by protective factors. However, the independence of the association between mortality and symptoms of depression indicates an increase in overall risk that is not completely mediated by these traditional risk factors or by loss of functionality for ADL.

This finding is corroborated by studies conducted in multiple countries which included broad control of confounding factors^24^, including one of individuals aged 35 to 70 years, a U.S. study that investigated individuals aged 45 years or older^25^, and a study conducted in Eastern Europe of individuals aged 45–69 years^26^. However, in addition to not having been conducted in middle-income countries, these studies included participants with a wider age range, unlike the present study, which enrolled exclusively 60 years-old or older adults, including centenarians. Characteristics associated with loneliness were also related to depression, such as older adults, males, and among those lacking a partner. Although some studies have found controversial results regarding gender and age, the analysis restricted to the elderly was able to detect a higher prevalence of depression in the elderly of a Spanish population^27^.

The present study also found a low prevalence of dependence for ADL at baseline. Nevertheless, about half of those with functional dependence died during the follow-up. Participants who experience deterioration of physical capacity inevitably need help from a caregiver, greater number of visits to health services, burdening individuals, families, and society. Use of the Barthel Index offers the advantage of allowing early detection of different domains of disability and its progression, in addition to predicting mortality^28^.

In this study, lower social support was not associated with mortality, in contrast to a previous investigation^29^. Veranópolis, a town built and largely populated by descendants of Italian immigrants, has several peculiar social characteristics: older adults live predominantly with relatives and interact closely with family, neighbors, and friends. These population-wide characteristics ensure that support networks extend well beyond the nuclear family, precluding the loneliness^30^. People who live with a partner, relatives, friends, or in communities may have their care needs better met, as may those with a broad-ranging social network. The observed negative correlations between the depression scale and various dimensions of social support, such as material, affective, emotional, information, and social interaction, can potentially be explained by these characteristics. This result has been previously documented^31^.

Some limitations deserve to be considered in the interpretation of the results. The study being conducted in a country city circumscribed the presence of a large population of Italian immigrants, like the Italians born in Brazil, which favored to detect the maintenance of proximity and healthier behaviors^32^ than those in the country of adoption. These characteristics were associated with the outcome of interest. Our study provided a glimpse in the centenarians, as this city has one of the highest life expectancy rates in the country. However, as they were outnumbered, limited statistical power prevented comparisons with other population-based studies^33^.

In conclusion, in the population of older adults observed in this cohort, symptoms of depression and loss of functionality for ADL are independent predictors of higher mortality. In addition to traditional risk factors, these aspects should be considered as part of the assessment and promotion of health in the older population.

## Supplementary Information

Supplementary Table 1.

## Data Availability

The data that support the findings of this study are available upon reasonable request from the corresponding author [SCF].
