# Supplementary material for: Mortality was predicted by depression and functional dependence in a cohort of elderly adults of Italian descent from southern Brazil
Source: Sci Rep. 2023 Apr 3;13:5448. doi: 10.1038/s41598-023-32617-1 (PMC10070406; doi:10.1038/s41598-023-32617-1)
Supplement: Supplementary file 1 — Supplementary Table 1. [file 41598_2023_32617_MOESM1_ESM.docx]

| Supplementary Table. Comparison of baseline characteristics of participants lost with those investigated in the follow-up | | |
| --- | --- | --- |
|  | n=882 (%) | n=115 (%) |
| Sex female | 544 (61.7) | 80 (69.6) |
| Age (years) |  |  |
| 60-69 | 335 (38.0) | 63 (54.8) |
| 70-79 | 357 (40.5) | 36 (31.3) |
| 80-89 | 155 (17.6) | 16 (13.9) |
| 90-102 | 35 (4.0) | 0 |
| Educational attainment (years) |  |  |
| ≥9 | 127 (14.4) | 16 (13.9) |
| 5-8 | 311 (35.3) | 31 (27.0) |
| 0-4 | 444 (50.3) | 68 (59.1) |
| Participation in group activities | 513 (58.2) | 72 (62.6) |
| Partner | 566 (64.2) | 69 (60.0) |
| Hypertension | 498 (56.5) | 71 (61.7) |
| Diabetes mellitus | 126 (14.3) | 19 (16.5) |
| Depression |  |  |
| No | 631 (71.5) | 85 (73.9) |
| Mild | 225 (25.5) | 26 (22.6) |
| Moderate/severe | 26 (3.0) | 4 (3.5) |
| Functionality for ADL |  |  |
| Independent | 740 (83.9) | 104 (90.4) |
| Dependent | 142 (16.1) | 11 (9.6) |
| Low social support | 709 (80.5) | 30 (26.1) |
| Heart disease | 286 (32.4) |  |
| Cancer | 51 (5.8) |  |
